# Application of the red-shifted channel rhodopsin Chrimson for the *Caenorhabditis elegans* cGAL bipartite system

**DOI:** 10.17912/2JGW-FJ52

**Published:** 2018-08-22

**Authors:** Mengyi Cao, Cynthia Chai, Jonathan Liu, Paul W Sternberg

**Affiliations:** 1 Division of Biology and Biological Engineering, California Institute of Technology

**Figure 1:**
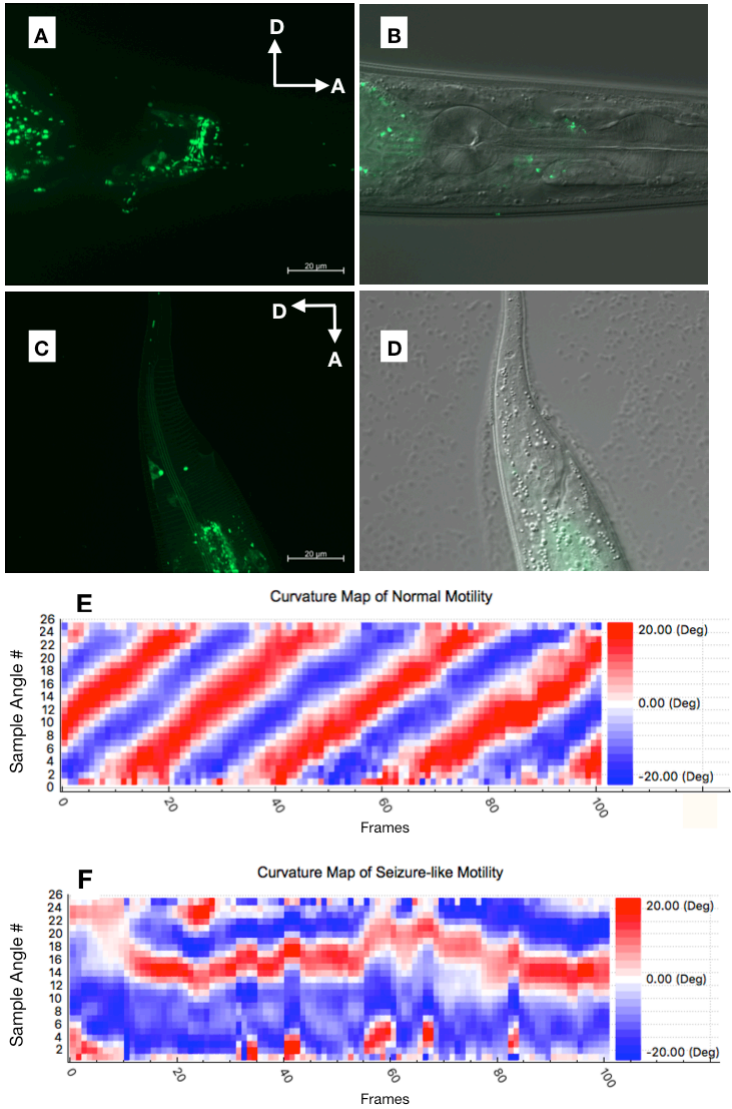
Pan-neuronal expression of Chrimson-GFP effector in adult hermaphrodite *C. elegans* in the head (A and B) and tail (C and D). cGAL pan-neuronal driver and Chrimson-GFP effector were integrated in *C. elegans* chromosome X(PS7946). Images were shown as GFP (A and C) and GFP/DIC microscopy overlay (B and D). Scale bar, 20 µm. (E-F) Curvature maps of a representative *C. elegans* with normal motility (E) and seizure-like motility (F). The animal exhibiting normal motility (from video 1) and seizure-like motility (from video 2) were each analyzed for time-lapse body curvature using WormLab 4 (2018 1.1). Curvatures at 26 positions along the centerline of the animal were sampled (y-axis) and monitored over 100 frames (x-axis) under each condition.

## Description

Channelrhodopsins are light-gated ion channels that serve as photoreceptors in photosynthetic microbes and have been applied as crucial optogenetic tools in genetic model organisms. When expressed in animals, they enable light-inducible control of ionic membrane permeability, which directly manipulates the activity of neurons expressing the protein. The application of channelrhodopsin-based optogenetics is particularly powerful when used in conjugation with the cGAL (GAL4-UAS) bipartite system (Wang *et al*., 2017). The mating of neuron-specific GAL4 driver lines to new channelrhodopsin effector lines could expand the genetic toolkit to perturb and manipulate neural circuits in the organism.

Blue light-gated channelrhodopsins have been widely used in *Caenorhabditis elegans* neurobiology but often have to be performed in *lite-1*(*ce314*) mutant backgrounds because short-wavelength blue light is an aversive cue in wild-type animals and directly affects *C. elegans* neuronal physiology. Previously, a red light-gated variant of channelrhodopsin, termed Chrimson, has been successfully applied in fruit fly and mice, and has recently been codon-optimized for use in *C. elegans* (Klapoetke *et al*., 2014; Schild and Glauser, 2015).

Here, we constructed a Chrimson (*15xUAS::chrimson::gfp*) cGAL effector line. We introduced the *UAS::chrimson::gfp* effector DNA construct as an extrachromosomal array into a previously published cGAL pan-neuronal driver line (PS6961 *syIs334*) and generated integrants on chromosome II (PS8023, *syIs503*) and chromosome V (PS8024, *syIs504*) (Table 1) via standard X-ray irradiation. We showed Chrimson-GFP expression in the *C. elegans* head and tail neurons (Fig. 1A-1D). We also showed that red light could induce a seizure-like motility phenotype in *C. elegans* expressing Chrimson-GFP in a pan-neuronal manner (videos), while the negative controls expressing only the effector, or without light induction showed regular motility as expected (Table 2). The body curvature maps from normal and seizure-like motilities showed distinct patterns (Fig. 1E and 1F). We report the effector construct of red-light-gated channelrhodopsin Chrimson as an addition to our cGAL toolkit, which could be widely used in future research to overcome the technical restrictions of blue light-gated channelrhodopsins in *C. elegans.*

**Videos:** Representative videos showing normal motility (video 1) and seizure-like motility (video 2) in a *C. elegans* adult hermaphrodite. Video 1: *C. elegans* co-expressing pan-neuronal driver and Chrimson-GFP effector (PS8026 *syIs504* V; *syIs334* X) was incubated with *all-trans retinal* (ATR) overnight and recorded motility without light stimulus, showing normal motility. Video 2: the same animal recorded in video 1 under the induction of intermittent red-light stimulus, showing seizure-like motility.

**Table 1:** A list of *C. elegans* strains used in this research.

**Table:** 

**Strain**	**Genotype**	**Description**	**Outcrossed**	**Reference**
PS6961	*syIs334* X	Integrated pan-neuronal driver (*Prab-3*)	2x	Wang and Liu *et al.*, 2017
PS7900	*syEx1626; syIs334* X	Chrimson extrachromosomal array in *Prab-3* driver background	NA	This research
PS7945	*syEx1626*	Chrimson extrachromosomal array in N2 background	NA	This research
PS7946	*syIs334 syIs497* X	*Prab-3* driver and Chrimson effector	4x	This research
PS8023	*syIs503* II	Chrimson effector	3x	This research
PS8024	*syIs504* V	Chrimson effector integrated on LGV	3x	This research
PS8025	*syIs503* II; *syIs334* X	Chrimson effector; *Prab-3* driver	3x	This research
PS8026	*syIs504* V; *syIs334* X	Chrimson effector; *Prab-3* driver	3x	This research
DA438	*bli-4(e937)* I; *rol6(e187)* II; *daf2(e1368) vab-7(e1562)* III; *unc-31(e928)* IV; *dpy-11(e224)* V; *lon2(e678)* X	Mapping strain	NA	Avery, 1993

**Table 2:** Red light induces seizure-like motility exclusively in animals co-express cGAL pan-neuronal driver and Chrimson effector. The numbers in the parentheses indicate the total numbers of animals screened in the behavioral assay, out of which 100% of the animals showed the same phenotype under each condition. (see videos for normal and seizure-like motility).

**Table:** 

**Driver**	**–**	**+**	**+**	**+**
**Effector**	**+**	**+**	**+**	**+**
**ATR**	**+**	**–**	**+**	**+**
**Light stimulus**	**+**	**+**	**–**	**+**
***syIs503***	Normal motility (11)	Normal motility (7)	Normal motility (11)	Seizure (10)
***syIs504***	Normal motility (11)	Normal motility (7)	Normal motility (11)	Seizure (11)

**Methods and Reagents**

*Molecular Cloning:* pJL057(*15xUAS::chrimson::gfp::let-858* 3’UTR) was created by inserting Chrimson via Gibson assembly into pHW394 cut with Kpn-I (NEB) upstream of the GFP. Chrimson was isolated via PCR using oJL122 (5’ttgGCTAGCgtcgacGGTAccaaaaATGGCCGAACTCATCTCATCG3’) and oJL123 (5’ CCTTTACTCATtttttctaccGGGACTGTATCCTCATCTTCCTC3’) from the template plasmid dg263 (Addgene #66101).

*DNA transformation in C. elegans:* Transgenic animals were generated using standard microinjection techniques, as found in Mello *et al*. Extrachromosomal arrays were integrated in the genome via X-ray irradiation.

*Strains:* PS7900 *syEx1626*; *syIs334* X was created by injecting pJL057(*15xUAS::chrimson::gfp::let-858* 3’UTR, 50 ng/uL), P*ofm-1*::gfp (50 ng/uL), and 150ng/uL of 1kb DNA ladder (NEB) into PS6961 (*Prab-3::GAL4* driver strain). PS7946 *syIs334* syIs497 X was created by integrating PS7900 and outcrossed 4x. PS8023 *syIs503* II and PS8024 *syIs504* V were obtained by integration of PS7945 *syEx1626* and outcrossed.

*Mapping:* To map the effector alleles to one of the six linkage groups in *C. elegans*, we used previously published methods by mating the effector lines with DA438 line (Walton *et al*., 2017 and Avery 1993).

*Microscopy and Fluorescence Screen:* The pan-neuronal expression of Chrimson-GFP was imaged under 100X magnification using a Zeiss Imager Z2 with Apotome 2.0 using Zen BLUE 2.3. Adult hermaphroditic worms were paralyzed on agarose pad (containing 3 mM levamisole and 2% agarose in M9 buffer). GFP images in Fig. 1A and 1C are maximum intensity projections.

*Optogenetics experiments: Escherichia coli* strain OP50 was grown in LB at 37° C overnight with aeration. The all-trans retinal (ATR, Sigma) was resuspended in ethanol to 100 mM and diluted 1:200 with the OP50 overnight culture to a final concentration of 500 µM. 100 uL of mixture of ATR and bacteria were seeded onto each NGM plates and air-dried for more than 30 min prior to the experiment. A total 12 animals from each line were picked onto three OP50 plates (4 animals per plate) with or without ATR and incubated overnight in the dark before the optogenetic experiments. The motility of worms was recorded for 2 min (7.5 frames per second) and analyzed using Wormlab Tracking system (MBF Biosciences). To induce depolarization, nematodes were exposed to pulses of red LED stimulus with 100 ms duration, 1100 ms interval length, and 100% intensity.
